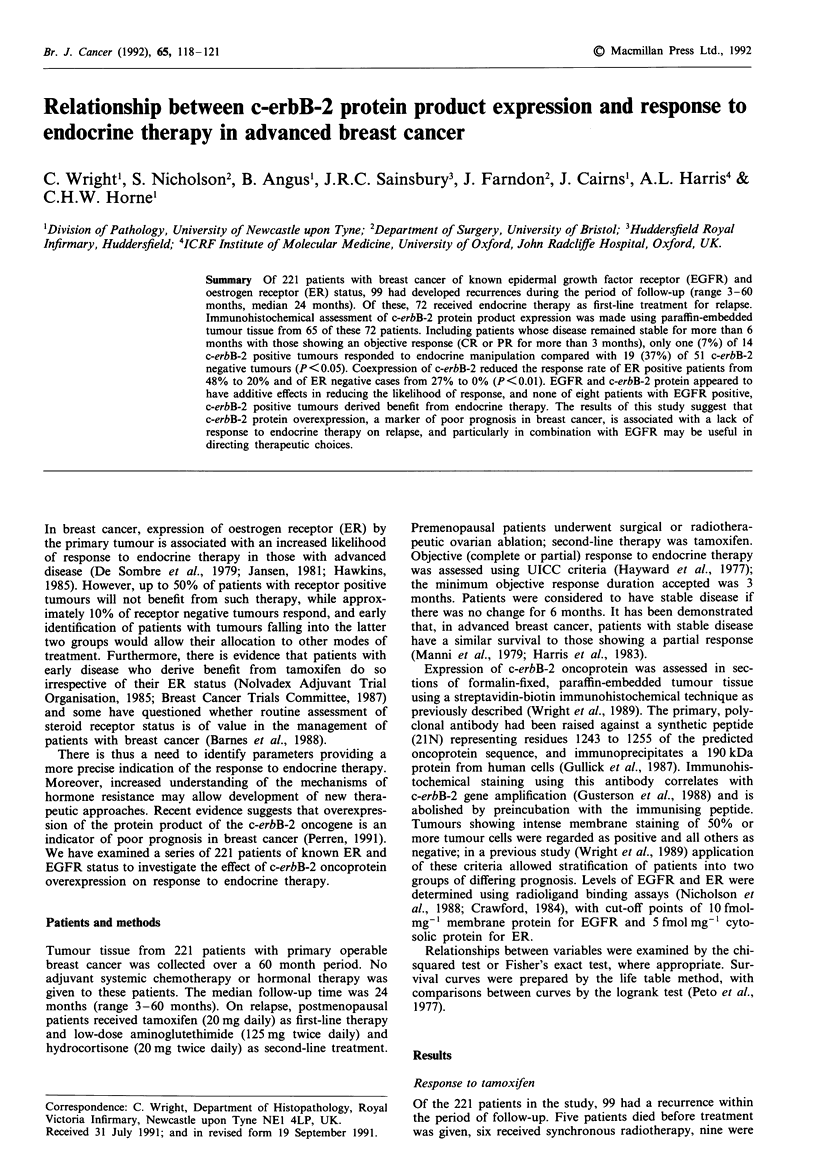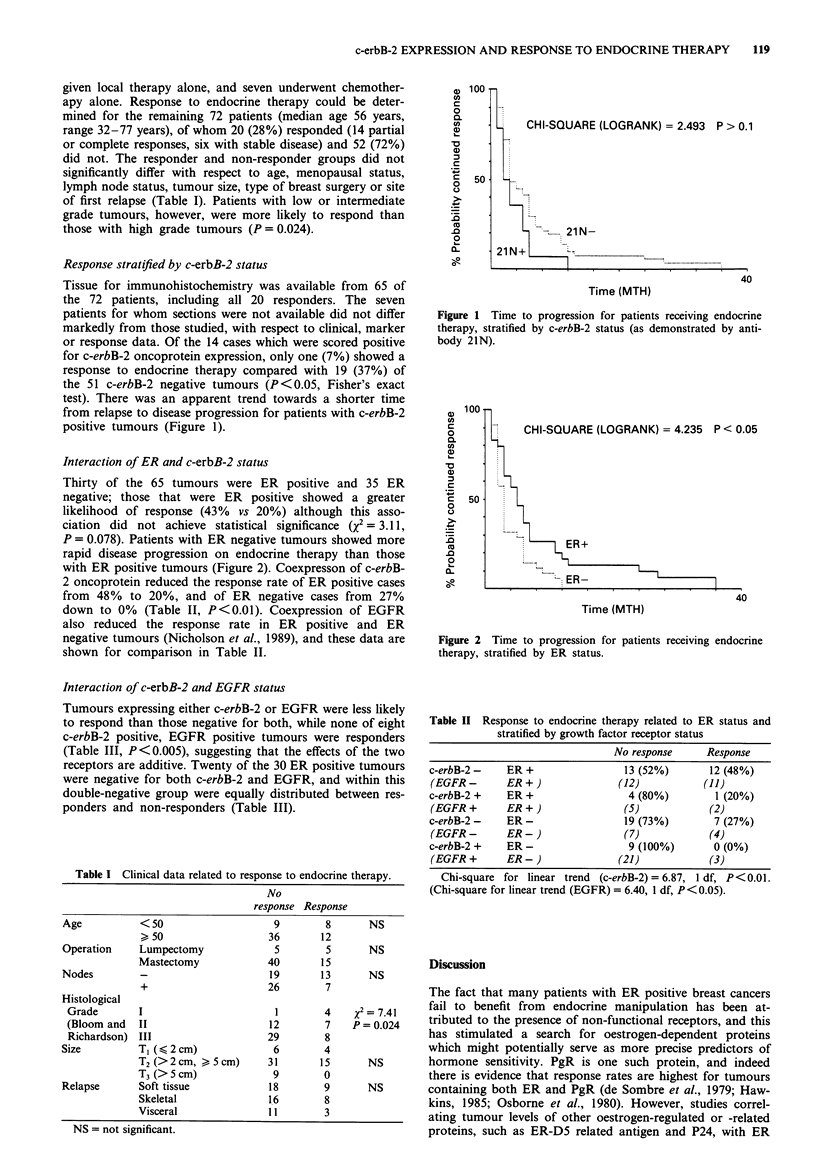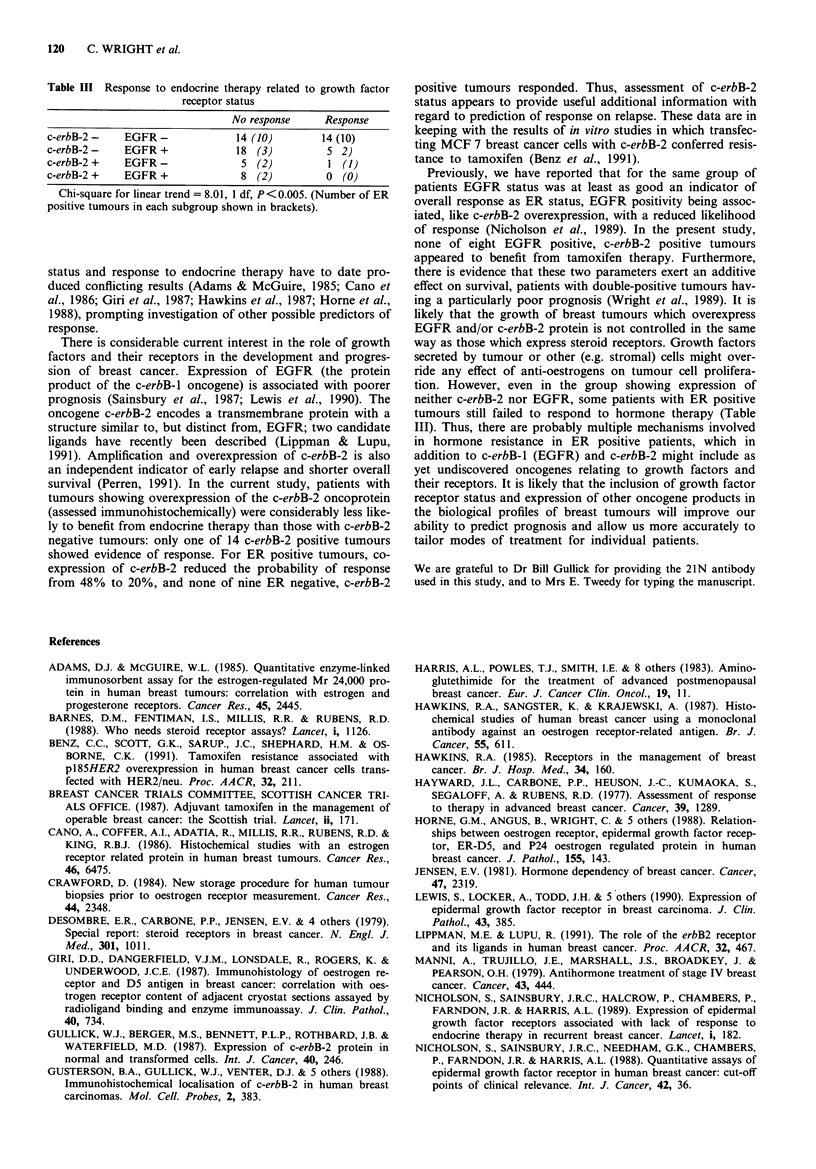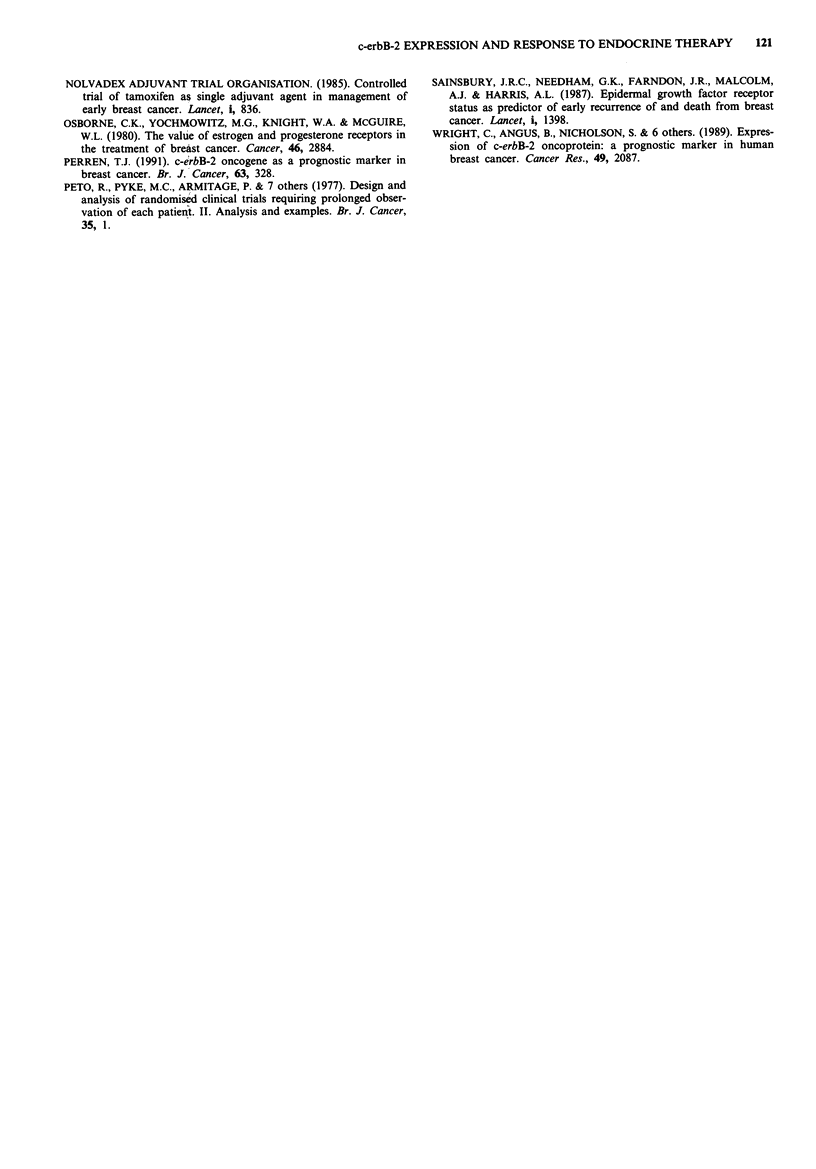# Relationship between c-erbB-2 protein product expression and response to endocrine therapy in advanced breast cancer.

**DOI:** 10.1038/bjc.1992.22

**Published:** 1992-01

**Authors:** C. Wright, S. Nicholson, B. Angus, J. R. Sainsbury, J. Farndon, J. Cairns, A. L. Harris, C. H. Horne

**Affiliations:** Division of Pathology, University of Newcastle upon Tyne, UK.

## Abstract

Of 221 patients with breast cancer of known epidermal growth factor receptor (EGFR) and oestrogen receptor (ER) status, 99 had developed recurrences during the period of follow-up (range 3-60 months, median 24 months). Of these, 72 received endocrine therapy as first-line treatment for relapse. Immunohistochemical assessment of c-erbB-2 protein product expression was made using paraffin-embedded tumour tissue from 65 of these 72 patients. Including patients whose disease remained stable for more than 6 months with those showing an objective response (CR or PR for more than 3 months), only one (7%) of 14 c-erbB-2 positive tumours responded to endocrine manipulation compared with 19 (37%) of 51 c-erbB-2 negative tumours (P less than 0.05). Coexpression of c-erbB-2 reduced the response rate of ER positive patients from 48% to 20% and of ER negative cases from 27% to 0% (P less than 0.01). EGFR and c-erbB-2 protein appeared to have additive effects in reducing the likelihood of response, and none of eight patients with EGFR positive, c-erbB-2 positive tumours derived benefit from endocrine therapy. The results of this study suggest that c-erbB-2 protein overexpression, a marker of poor prognosis in breast cancer, is associated with a lack of response to endocrine therapy on relapse, and particularly in combination with EGFR may be useful in directing therapeutic choices.


 © Macmillan Press Ltd., 1992

### Relationship between c-erbB-2 protein product expression and response to endocrine therapy in advanced breast cancer

C. Wright[1], S. Nicholson[2], B. Angus[1], J.R.C. Sainsbury[3], J. Farndon[2], J. Cairns[1], A.L. Harris[4] & C.H.W. Horne[1]

[1]*Division of Pathology, University of Newcastle upon Tyne;* [2]*Department of Surgery, University of Bristol;* [3]*Huddersfield Royal Infirmary, Huddersfield;* [4]*ICRF Institute of Molecular Medicine, University of Oxford, John Radcliffe Hospital, Oxford, UK.*

**Summary** Of 221 patients with breast cancer of known epidermal growth factor receptor (EGFR) and oestrogen receptor (ER) status, 99 had developed recurrences during the period of follow-up (range 3-60 months, median 24 months). Of these, 72 received endocrine therapy as first-line treatment for relapse. Immunohistochemical assessment of c-*erb*B-2 protein product expression was made using paraffin-embedded tumour tissue from 65 of these 72 patients. Including patients whose disease remained stable for more than 6 months with those showing an objective response (CR or PR for more than 3 months), only one (7%) of 14 c-*erb*B-2 positive tumours responded to endocrine manipulation compared with 19 (37%) of 51 c-*erb*B-2 negative tumours ($P < 0.05$). Coexpression of c-*erb*B-2 reduced the response rate of ER positive patients from 48% to 20% and of ER negative cases from 27% to 0% ($P < 0.01$). EGFR and c-*erb*B-2 protein appeared to have additive effects in reducing the likelihood of response, and none of eight patients with EGFR positive, c-*erb*B-2 positive tumours derived benefit from endocrine therapy. The results of this study suggest that c-*erb*B-2 protein overexpression, a marker of poor prognosis in breast cancer, is associated with a lack of response to endocrine therapy on relapse, and particularly in combination with EGFR may be useful in directing therapeutic choices.

In breast cancer, expression of oestrogen receptor (ER) by the primary tumour is associated with an increased likelihood of response to endocrine therapy in those with advanced disease (De Sombre *et al.*, 1979; Jansen, 1981; Hawkins, 1985). However, up to 50% of patients with receptor positive tumours will not benefit from such therapy, while approximately 10% of receptor negative tumours respond, and early identification of patients with tumours falling into the latter two groups would allow their allocation to other modes of treatment. Furthermore, there is evidence that patients with early disease who derive benefit from tamoxifen do so irrespective of their ER status (Nolvadex Adjuvant Trial Organisation, 1985; Breast Cancer Trials Committee, 1987) and some have questioned whether routine assessment of steroid receptor status is of value in the management of patients with breast cancer (Barnes *et al.*, 1988).

There is thus a need to identify parameters providing a more precise indication of the response to endocrine therapy. Moreover, increased understanding of the mechanisms of hormone resistance may allow development of new therapeutic approaches. Recent evidence suggests that overexpression of the protein product of the c-*erb*B-2 oncogene is an indicator of poor prognosis in breast cancer (Perren, 1991). We have examined a series of 221 patients of known ER and EGFR status to investigate the effect of c-*erb*B-2 oncoprotein overexpression on response to endocrine therapy.

### Patients and methods

Tumour tissue from 221 patients with primary operable breast cancer was collected over a 60 month period. No adjuvant systemic chemotherapy or hormonal therapy was given to these patients. The median follow-up time was 24 months (range 3-60 months). On relapse, postmenopausal patients received tamoxifen (20 mg daily) as first-line therapy and low-dose aminoglutethimide (125 mg twice daily) and hydrocortisone (20 mg twice daily) as second-line treatment. Premenopausal patients underwent surgical or radiotherapeutic ovarian ablation; second-line therapy was tamoxifen. Objective (complete or partial) response to endocrine therapy was assessed using UICC criteria (Hayward *et al.*, 1977); the minimum objective response duration accepted was 3 months. Patients were considered to have stable disease if there was no change for 6 months. It has been demonstrated that, in advanced breast cancer, patients with stable disease have a similar survival to those showing a partial response (Manni *et al.*, 1979; Harris *et al.*, 1983).

Expression of c-*erb*B-2 oncoprotein was assessed in sections of formalin-fixed, paraffin-embedded tumour tissue using a streptavidin-biotin immunohistochemical technique as previously described (Wright *et al.*, 1989). The primary, polyclonal antibody had been raised against a synthetic peptide (21N) representing residues 1243 to 1255 of the predicted oncoprotein sequence, and immunoprecipitates a 190 kDa protein from human cells (Gullick *et al.*, 1987). Immunohistochemical staining using this antibody correlates with c-*erb*B-2 gene amplification (Gusterson *et al.*, 1988) and is abolished by preincubation with the immunising peptide. Tumours showing intense membrane staining of 50% or more tumour cells were regarded as positive and all others as negative; in a previous study (Wright *et al.*, 1989) application of these criteria allowed stratification of patients into two groups of differing prognosis. Levels of EGFR and ER were determined using radioligand binding assays (Nicholson *et al.*, 1988; Crawford, 1984), with cut-off points of 10 fmol $mg^{-1}$ membrane protein for EGFR and 5 fmol $mg^{-1}$ cytosolic protein for ER.

Relationships between variables were examined by the chi-squared test or Fisher's exact test, where appropriate. Survival curves were prepared by the life table method, with comparisons between curves by the logrank test (Peto *et al.*, 1977).

### Results

### *Response to tamoxifen*

Of the 221 patients in the study, 99 had a recurrence within the period of follow-up. Five patients died before treatment was given, six received synchronous radiotherapy, nine were

Correspondence: C. Wright, Department of Histopathology, Royal Victoria Infirmary, Newcastle upon Tyne NE1 4LP, UK.
Received 31 July 1991; and in revised form 19 September 1991.

given local therapy alone, and seven underwent chemotherapy alone. Response to endocrine therapy could be determined for the remaining 72 patients (median age 56 years, range 32–77 years), of whom 20 (28%) responded (14 partial or complete responses, six with stable disease) and 52 (72%) did not. The responder and non-responder groups did not significantly differ with respect to age, menopausal status, lymph node status, tumour size, type of breast surgery or site of first relapse (Table I). Patients with low or intermediate grade tumours, however, were more likely to respond than those with high grade tumours ($P = 0.024$).

### *Response stratified by c-*erb*B-2 status*

Tissue for immunohistochemistry was available from 65 of the 72 patients, including all 20 responders. The seven patients for whom sections were not available did not differ markedly from those studied, with respect to clinical, marker or response data. Of the 14 cases which were scored positive for c-*erb*B-2 oncoprotein expression, only one (7%) showed a response to endocrine therapy compared with 19 (37%) of the 51 c-*erb*B-2 negative tumours ($P<0.05$, Fisher's exact test). There was an apparent trend towards a shorter time from relapse to disease progression for patients with c-*erb*B-2 positive tumours (Figure 1).

### *Interaction of ER and c-*erb*B-2 status*

Thirty of the 65 tumours were ER positive and 35 ER negative; those that were ER positive showed a greater likelihood of response (43% *vs* 20%) although this association did not achieve statistical significance ($\chi^2 = 3.11$, $P = 0.078$). Patients with ER negative tumours showed more rapid disease progression on endocrine therapy than those with ER positive tumours (Figure 2). Coexpresson of c-*erb*B-2 oncoprotein reduced the response rate of ER positive cases from 48% to 20%, and of ER negative cases from 27% down to 0% (Table II, $P<0.01$). Coexpression of EGFR also reduced the response rate in ER positive and ER negative tumours (Nicholson *et al.*, 1989), and these data are shown for comparison in Table II.

### *Interaction of c-*erb*B-2 and EGFR status*

Tumours expressing either c-*erb*B-2 or EGFR were less likely to respond than those negative for both, while none of eight c-*erb*B-2 positive, EGFR positive tumours were responders (Table III, $P<0.005$), suggesting that the effects of the two receptors are additive. Twenty of the 30 ER positive tumours were negative for both c-*erb*B-2 and EGFR, and within this double-negative group were equally distributed between responders and non-responders (Table III).

**Table I** Clinical data related to response to endocrine therapy.

| | | No response | Response | |
|---|---|---|---|---|
| Age | <50 | 9 | 8 | NS |
| | ⩾50 | 36 | 12 | |
| Operation | Lumpectomy | 5 | 5 | NS |
| | Mastectomy | 40 | 15 | |
| Nodes | − | 19 | 13 | NS |
| | + | 26 | 7 | |
| Histological Grade | I | 1 | 4 | $\chi^2 = 7.41$ |
| (Bloom and | II | 12 | 7 | $P = 0.024$ |
| Richardson) | III | 29 | 8 | |
| Size | $T_1$ (⩽2 cm) | 6 | 4 | |
| | $T_2$ (>2 cm, ⩾5 cm) | 31 | 15 | NS |
| | $T_3$ (>5 cm) | 9 | 0 | |
| Relapse | Soft tissue | 18 | 9 | NS |
| | Skeletal | 16 | 8 | |
| | Visceral | 11 | 3 | |

NS = not significant.

**Figure 1** Time to progression for patients receiving endocrine therapy, stratified by c-*erb*B-2 status (as demonstrated by antibody 21N).

**Figure 2** Time to progression for patients receiving endocrine therapy, stratified by ER status.

**Table II** Response to endocrine therapy related to ER status and stratified by growth factor receptor status

| | | No response | Response |
|---|---|---|---|
| c-*erb*B-2 − | ER + | 13 (52%) | 12 (48%) |
| *(EGFR −* | *ER + )* | *(12)* | *(11)* |
| c-*erb*B-2 + | ER + | 4 (80%) | 1 (20%) |
| *(EGFR +* | *ER + )* | *(5)* | *(2)* |
| c-*erb*B-2 − | ER − | 19 (73%) | 7 (27%) |
| *(EGFR −* | *ER − )* | *(7)* | *(4)* |
| c-*erb*B-2 + | ER − | 9 (100%) | 0 (0%) |
| *(EGFR +* | *ER − )* | *(21)* | *(3)* |

Chi-square for linear trend (c-*erb*B-2) = 6.87, 1 df, $P<0.01$. (Chi-square for linear trend (EGFR) = 6.40, 1 df, $P<0.05$).

### Discussion

The fact that many patients with ER positive breast cancers fail to benefit from endocrine manipulation has been attributed to the presence of non-functional receptors, and this has stimulated a search for oestrogen-dependent proteins which might potentially serve as more precise predictors of hormone sensitivity. PgR is one such protein, and indeed there is evidence that response rates are highest for tumours containing both ER and PgR (de Sombre *et al.*, 1979; Hawkins, 1985; Osborne *et al.*, 1980). However, studies correlating tumour levels of other oestrogen-regulated or -related proteins, such as ER-D5 related antigen and P24, with ER

**Table III** Response to endocrine therapy related to growth factor receptor status

| | | *No response* | *Response* |
|---|---|---|---|
| c-*erb*B-2 – | EGFR – | 14 *(10)* | 14 (10) |
| c-*erb*B-2 – | EGFR + | 18 *(3)* | 5 *2)* |
| c-*erb*B-2 + | EGFR – | 5 *(2)* | 1 *(1)* |
| c-*erb*B-2 + | EGFR + | 8 *(2)* | 0 *(0)* |

Chi-square for linear trend = 8.01, 1 df, $P < 0.005$. (Number of ER positive tumours in each subgroup shown in brackets).

status and response to endocrine therapy have to date produced conflicting results (Adams & McGuire, 1985; Cano *et al.*, 1986; Giri *et al.*, 1987; Hawkins *et al.*, 1987; Horne *et al.*, 1988), prompting investigation of other possible predictors of response.

There is considerable current interest in the role of growth factors and their receptors in the development and progression of breast cancer. Expression of EGFR (the protein product of the c-*erb*B-1 oncogene) is associated with poorer prognosis (Sainsbury *et al.*, 1987; Lewis *et al.*, 1990). The oncogene c-*erb*B-2 encodes a transmembrane protein with a structure similar to, but distinct from, EGFR; two candidate ligands have recently been described (Lippman & Lupu, 1991). Amplification and overexpression of c-*erb*B-2 is also an independent indicator of early relapse and shorter overall survival (Perren, 1991). In the current study, patients with tumours showing overexpression of the c-*erb*B-2 oncoprotein (assessed immunohistochemically) were considerably less likely to benefit from endocrine therapy than those with c-*erb*B-2 negative tumours: only one of 14 c-*erb*B-2 positive tumours showed evidence of response. For ER positive tumours, co-expression of c-*erb*B-2 reduced the probability of response from 48% to 20%, and none of nine ER negative, c-*erb*B-2 positive tumours responded. Thus, assessment of c-*erb*B-2 status appears to provide useful additional information with regard to prediction of response on relapse. These data are in keeping with the results of *in vitro* studies in which transfecting MCF 7 breast cancer cells with c-*erb*B-2 conferred resistance to tamoxifen (Benz *et al.*, 1991).

Previously, we have reported that for the same group of patients EGFR status was at least as good an indicator of overall response as ER status, EGFR positivity being associated, like c-*erb*B-2 overexpression, with a reduced likelihood of response (Nicholson *et al.*, 1989). In the present study, none of eight EGFR positive, c-*erb*B-2 positive tumours appeared to benefit from tamoxifen therapy. Furthermore, there is evidence that these two parameters exert an additive effect on survival, patients with double-positive tumours having a particularly poor prognosis (Wright *et al.*, 1989). It is likely that the growth of breast tumours which overexpress EGFR and/or c-*erb*B-2 protein is not controlled in the same way as those which express steroid receptors. Growth factors secreted by tumour or other (e.g. stromal) cells might override any effect of anti-oestrogens on tumour cell proliferation. However, even in the group showing expression of neither c-*erb*B-2 nor EGFR, some patients with ER positive tumours still failed to respond to hormone therapy (Table III). Thus, there are probably multiple mechanisms involved in hormone resistance in ER positive patients, which in addition to c-*erb*B-1 (EGFR) and c-*erb*B-2 might include as yet undiscovered oncogenes relating to growth factors and their receptors. It is likely that the inclusion of growth factor receptor status and expression of other oncogene products in the biological profiles of breast tumours will improve our ability to predict prognosis and allow us more accurately to tailor modes of treatment for individual patients.

We are grateful to Dr Bill Gullick for providing the 21N antibody used in this study, and to Mrs E. Tweedy for typing the manuscript.

### References

ADAMS, D.J. & MCGUIRE, W.L. (1985). Quantitative enzyme-linked immunosorbent assay for the estrogen-regulated Mr 24,000 protein in human breast tumours: correlation with estrogen and progesterone receptors. *Cancer Res.*, **45**, 2445.

BARNES, D.M., FENTIMAN, I.S., MILLIS, R.R. & RUBENS, R.D. (1988). Who needs steroid receptor assays? *Lancet*, **i**, 1126.

BENZ, C.C., SCOTT, G.K., SARUP., J.C., SHEPHARD, H.M. & OSBORNE, C.K. (1991). Tamoxifen resistance associated with p185*HER2* overexpression in human breast cancer cells transfected with HER2/neu. *Proc. AACR*, **32**, 211.

BREAST CANCER TRIALS COMMITTEE, SCOTTISH CANCER TRIALS OFFICE. (1987). Adjuvant tamoxifen in the management of operable breast cancer: the Scottish trial. *Lancet*, **ii**, 171.

CANO, A., COFFER, A.I., ADATIA, R., MILLIS, R.R., RUBENS, R.D. & KING, R.B.J. (1986). Histochemical studies with an estrogen receptor related protein in human breast tumours. *Cancer Res.*, **46**, 6475.

CRAWFORD, D. (1984). New storage procedure for human tumour biopsies prior to oestrogen receptor measurement. *Cancer Res.*, **44**, 2348.

DESOMBRE, E.R., CARBONE, P.P., JENSEN, E.V. & 4 others (1979). Special report: steroid receptors in breast cancer. *N. Engl. J. Med.*, **301**, 1011.

GIRI, D.D., DANGERFIELD, V.J.M., LONSDALE, R., ROGERS, K. & UNDERWOOD, J.C.E. (1987). Immunohistology of oestrogen receptor and D5 antigen in breast cancer: correlation with oestrogen receptor content of adjacent cryostat sections assayed by radioligand binding and enzyme immunoassay. *J. Clin. Pathol.*, **40**, 734.

GULLICK, W.J., BERGER, M.S., BENNETT, P.L.P., ROTHBARD, J.B. & WATERFIELD, M.D. (1987). Expression of c-*erb*B-2 protein in normal and transformed cells. *Int. J. Cancer*, **40**, 246.

GUSTERSON, B.A., GULLICK, W.J., VENTER, D.J. & 5 others (1988). Immunohistochemical localisation of c-*erb*B-2 in human breast carcinomas. *Mol. Cell. Probes*, **2**, 383.

HARRIS, A.L., POWLES, T.J., SMITH, I.E. & 8 others (1983). Aminoglutethimide for the treatment of advanced postmenopausal breast cancer. *Eur. J. Cancer Clin. Oncol.*, **19**, 11.

HAWKINS, R.A., SANGSTER, K. & KRAJEWSKI, A. (1987). Histochemical studies of human breast cancer using a monoclonal antibody against an oestrogen receptor-related antigen. *Br. J. Cancer*, **55**, 611.

HAWKINS, R.A. (1985). Receptors in the management of breast cancer. *Br. J. Hosp. Med.*, **34**, 160.

HAYWARD, J.L., CARBONE, P.P., HEUSON, J.-C., KUMAOKA, S., SEGALOFF, A. & RUBENS, R.D. (1977). Assessment of response to therapy in advanced breast cancer. *Cancer*, **39**, 1289.

HORNE, G.M., ANGUS, B., WRIGHT, C. & 5 others (1988). Relationships between oestrogen receptor, epidermal growth factor receptor, ER-D5, and P24 oestrogen regulated protein in human breast cancer. *J. Pathol.*, **155**, 143.

JENSEN, E.V. (1981). Hormone dependency of breast cancer. *Cancer*, **47**, 2319.

LEWIS, S., LOCKER, A., TODD, J.H. & 5 others (1990). Expression of epidermal growth factor receptor in breast carcinoma. *J. Clin. Pathol.*, **43**, 385.

LIPPMAN, M.E. & LUPU, R. (1991). The role of the *erb*B2 receptor and its ligands in human breast cancer. *Proc. AACR*, **32**, 467.

MANNI, A., TRUJILLO, J.E., MARSHALL, J.S., BROADKEY, J. & PEARSON, O.H. (1979). Antihormone treatment of stage IV breast cancer. *Cancer*, **43**, 444.

NICHOLSON, S., SAINSBURY, J.R.C., HALCROW, P., CHAMBERS, P., FARNDON, J.R. & HARRIS, A.L. (1989). Expression of epidermal growth factor receptors associated with lack of response to endocrine therapy in recurrent breast cancer. *Lancet*, **i**, 182.

NICHOLSON, S., SAINSBURY, J.R.C., NEEDHAM, G.K., CHAMBERS, P., FARNDON, J.R. & HARRIS, A.L. (1988). Quantitative assays of epidermal growth factor receptor in human breast cancer: cut-off points of clinical relevance. *Int. J. Cancer*, **42**, 36.

NOLVADEX ADJUVANT TRIAL ORGANISATION. (1985). Controlled trial of tamoxifen as single adjuvant agent in management of early breast cancer. *Lancet*, **i**, 836.

OSBORNE, C.K., YOCHMOWITZ, M.G., KNIGHT, W.A. & MCGUIRE, W.L. (1980). The value of estrogen and progesterone receptors in the treatment of breast cancer. *Cancer*, **46**, 2884.

PERREN, T.J. (1991). c-*erb*B-2 oncogene as a prognostic marker in breast cancer. *Br. J. Cancer*, **63**, 328.

PETO, R., PYKE, M.C., ARMITAGE, P. & 7 others (1977). Design and analysis of randomised clinical trials requiring prolonged observation of each patient. II. Analysis and examples. *Br. J. Cancer*, **35**, 1.

SAINSBURY, J.R.C., NEEDHAM, G.K., FARNDON, J.R., MALCOLM, A.J. & HARRIS, A.L. (1987). Epidermal growth factor receptor status as predictor of early recurrence of and death from breast cancer. *Lancet*, **i**, 1398.

WRIGHT, C., ANGUS, B., NICHOLSON, S. & 6 others. (1989). Expression of c-*erb*B-2 oncoprotein: a prognostic marker in human breast cancer. *Cancer Res.*, **49**, 2087.